# Adverse Effects of Amoxicillin for Acute Lower Respiratory Tract Infection in Primary Care: Secondary and Subgroup Analysis of a Randomised Clinical Trial

**DOI:** 10.3390/antibiotics6040036

**Published:** 2017-12-13

**Authors:** Meera Tandan, Akke Vellinga, Robin Bruyndonckx, Paul Little, Theo Verheij, Chris C Butler, Herman Goossens, Samuel Coenen

**Affiliations:** 1 Discipline of General Practice, School of Medicine, National University of Ireland Galway (NUIG), Galway, Ireland; akke.vellinga@nuigalway.ie; 2 Discipline of Bacteriology, School of Medicine, National University of Ireland Galway (NUIG), Galway, Ireland; 3 Interuniversity Institute for Biostatistics and statistical Bioinformatics (I-BioStat), Hasselt University, 3500 Hasselt, Belgium; robin.bruyndonckx@uhasselt.be; 4 Primary Care and Population Science, University of Southampton, Southampton SO17 1BJ, UK; p.little@soton.ac.uk; 5 Julius Center for Health Science and Primary Care, University Medical Center Utrecht, 3508 Utrecht, GA, The Netherlands; t.j.m.verheij@umcutrecht.nl; 6 The Nuffield Department of Primary Care Health Sciences, University of Oxford, Oxford OX2 6GG, UK; christopher.butler@phc.ox.ac.uk; 7 School of Medicine, Cardiff University, Cardiff CF14 4XN, UK; 8 Laboratory Medical Microbiology, Vaccine & Infectious Disease Institute (VAXINFECTIO), University of Antwerp, 2610 Antwerp, Belgium; Herman.Goossens@uza.be (H.G.); samuel.coenen@uantwerpen.be (S.C.); 9 Centre for General Practice, Department of Primary and Interdisciplinary Care (ELIZA), University of Antwerp, 2610 Antwerp, Belgium; 10 Department of Epidemiology and Social Medicine (ESOC), University of Antwerp, 2610 Antwerp, Belgium

**Keywords:** adverse effects, antibiotics, respiratory infections, randomized controlled trial, primary care, subgroup analysis

## Abstract

A European placebo-controlled trial of antibiotic treatment for lower respiratory tract infection (LRTI) conducted in 16 primary care practices networks recruited participants between November 2007 and April 2010, and found adverse events (AEs) occurred more often in patients prescribed amoxicillin compared to placebo. This secondary analysis explores the causal relationship and estimates specific AEs (diarrhoea, nausea, rash) due to amoxicillin treatment for LRTI, and if any subgroup is at increased risk of any or a specific AE. A total of 2061 patients were randomly assigned to amoxicillin (1038) and placebo (1023); 595 (28%) were 60 and older. A significantly higher proportion of any AEs (diarrhoea or nausea or rash) (OR = 1.31, 95% CI 1.05–1.64, number needed to harm (NNH) = 24) and of diarrhoea (OR 1.43 95% CI 1.08–1.90, NNH = 29) was reported in the amoxicillin group during the first week after randomisation. Subgroup analysis showed rash was significantly more often reported in males prescribed amoxicillin (interaction term 3.72 95% CI 1.22–11.36; OR of amoxicillin in males 2.79 (95% CI 1.08–7.22). No other subgroup at higher risk was identified. Although the study was not powered for subgroup analysis, this analysis suggests that most patients are likely to be equally harmed when prescribed antibiotics.

## 1. Introduction

Lower respiratory tract infection (LRTI) is the most common reason for consulting a general practitioner (GP) [1,2]. LRTIs are often treated with antibiotics, even though this is not generally supported by guidelines and recommendations [2,3,4,5,6]. Many trials and observational studies have found no or little benefit of antibiotic treatment for an acute cough [7]. If an antibiotic is prescribed, amoxicillin is the recommended first-line treatment for LRTI [8]. Amoxicillin is the most commonly used broad-spectrum penicillin accounting for an average 40% of the total outpatient antibiotic use in Europe [8,9,10].

All medications have known adverse events (AEs) and antibiotics are no exceptions [11]. Although most antibiotics are generally considered safe, and most AEs are moderate to mild, some antibiotics have been associated with life-threatening AEs [12]. AEs are generally poorly reported in trials, and their true incidence is thought to be much higher than reported in trials [13]. In primary care, a shared decision consultation should include both the benefits and potential harms of the (antibiotic) treatment prescribed [14].

The European multicentre randomised placebo-controlled trial (RCT) of amoxicillin for LRTI in adults in primary care was performed by the GRACE (Genomics to combat Resistance against Antibiotics in Community-acquired LRTI in Europe; http://www.grace-lrti.org) Network of Excellence. The GRACE trial identified significantly more AEs (diarrhoea or nausea or rash) in the amoxicillin group compared to the placebo group (AEs in week one and week two after antibiotic administration) [15]. However, it was not clear whether this applies to each specific AE, or whether particular subgroups of patients suffer more AEs than others. To better inform primary care clinicians and their patients, this secondary analysis of the GRACE trial aims to provide estimates of any and each specific AE (diarrhoea, nausea, and rash) of amoxicillin, and identify subgroups of patients that are more at risk for any or a specific AE.

## 2. Results

### 2.1. Participant’s Distribution by Subgroups

A total of 2061 patients were recruited and randomly assigned to amoxicillin (1038) and to placebo (1023). Of the total, 595 (28%) were 60 years and older, 836 (40%) were males, 579 (28%) current smokers, and 470 (23%) reported symptoms of anxiety/depression on the day of consultation. Regarding the medication history, 283 (14%) had antibiotic treatment in previous 6 months, 1056 (51%) reported over-the-counter (OTC) treatment before consulting a general practitioner, 1342 (65%) were prescribed treatment other than study medication during their consultation. These other medications included 479 (23%) antihypertensive/diuretics, 201 (10%) oral bronchodilators, 171 (8%) steroids, 202 (10%) antidepressant/benzodiazepines, 174 (8%) non-steroidal anti-inflammatory drugs, and 451 (22%) influenza vaccines.

### 2.2. Subgroup Analysis of Adverse Events

For the whole cohort, at the end of week one, a significantly higher proportion of any AE (diarrhoea or nausea or rash) was reported in the amoxicillin group compared to placebo (OR = 1.31, 95% CI 1.05–1.64) (Table 1). The number needed to harm (NNH) was 24, i.e., on average, for every 24 patients receiving amoxicillin, one additional patient had reported any AE due to the antibiotic at the end of week one. Any AE was reported significantly more often in patients ever being a smoker (OR = 1.41, 95% CI 1.04–1.90), and patients using OTC treatment before the consultation (OR = 1.44, 95% CI 1.09–1.91), but the interaction terms were not statistically significant. In patients with depression/anxiety on the day of consultation (OR = 1.49, 95% CI 0.99–2.22) and in those on any medication other than study medication (OR = 1.29, 95% CI 0.98–1.69) the odds ratios were borderline significant, but the interaction terms were not significant. This indicates that compared to the whole cohort, no subgroups were at higher risk of any AE (diarrhoea or nausea or rash) due to amoxicillin.

Analysing each specific AE, diarrhoea was present significantly more often among patients in the amoxicillin group, compared to those in the placebo group (OR 1.43 CI 1.08–1.90, Table 2) (NNH: 29). Diarrhoea was significantly more often reported by patients 60 years and over (OR 1.97, 95% CI 1.09–3.54), current smokers (OR 2.07, 95% CI 1.15–3.76), ever being a smoker (OR 1.79, 95% CI 1.21–2.65), on OTC treatment before their consultation (OR 1.76 95% CI 1.23–2.53), and on antihypertensive/diuretics (OR 2.27, 95% CI 1.27–4.05). However, the interaction terms were not significant.

Nausea was not associated with amoxicillin treatment for either the whole cohort or any subgroup of patients (Table 3). Rash was only significantly more often reported by males (interaction term 3.72, *p* = 0.021; odds ratio in males 2.79 (95% CI 1.08–7.22) (Table 4).

## 3. Discussion

### 3.1. Summary

To the authors’ knowledge, this is the first subgroup analysis of any and specific AEs reported in RCTs of antibiotics for LRTI. Diarrhoea was significantly more likely to be reported in the amoxicillin group compared to the placebo group. No specific subgroups were at higher risk of any or a specific AE due to amoxicillin, apart from males in the amoxicillin group reporting rash significantly more often.

### 3.2. Strengths and Limitations

Our results are based on data from the largest RCT of antibiotics for acute LRTI in general practice to date [15]. Its primary objective was not identifying the incidence of AEs. RCTs, such as the GRACE trial, are not always prospectively powered for subgroup analysis of AEs [16]. Accordingly, subgroup analyses with multiple comparisons are often underpowered, with a greater risk of the false negative results (type II error). Large sample sizes are needed for robust subgroup analysis, which may only be possible by combining trial results in a meta-analysis [17].

### 3.3. Comparison with Existing Literature

Reporting guidelines on RCTs indicate that more details on AEs of medication should be documented and reported to the concerned authority [18]. However, AEs occurring during a trial are often underreported, in particular, when reporting results in trial publications. Underreporting may be a result of poor monitoring, missing data, or unclear case definitions [19]. An important consequence of underreporting of AEs is a misinterpretation of the intervention’s effects, particularly its harms [20]. Although the GRACE trial captured AEs from the study medication, the reported AEs were limited to diarrhoea, nausea, and rash. A review paper identified that candidiasis was significantly associated with amoxicillin use [13], and patients treated with amoxicillin were twice as likely, compared to placebo, to report diarrhoea [13,19]. As in the previously published paper from GRACE trial [15], the current study showed significantly more AEs (diarrhoea or nausea or rash) in amoxicillin group compared to placebo. The calculation of any AEs in the previous paper covered the first two weeks after the antibiotic was prescribed, however, this paper reports on any AEs in the first week while patients were taking antibiotics.

This study also showed a higher risk overall of diarrhoea in the amoxicillin group compared to the placebo group. Even though diarrhoea was more often reported in the treatment group for patients 60 years and older, smokers, patients taking OTC treatment before consulting a GP, patients on antihypertensive or non-steroidal anti-inflammatory drugs and those who received an influenza vaccine, no particular subgroup was at higher risk of AEs.

The presented analysis showed that males in the amoxicillin group reported rash significantly more often compared to males in the placebo group. Skin reactions were also associated with amoxicillin use [21]. A borderline significant interaction term was observed for patients reporting anxiety/depression on the day of the consultation. These (borderline) significant results may be due to multiple testing, and with more conservative *p*-values, and these results would not be considered significant [22]. Sensitivity analysis did not alter our conclusions.

## 4. Materials and Methods

### 4.1. Study Design and Patients

The GRACE trial was performed in 16 primary care research networks in 12 European countries. Details of the study design, patient inclusion, and recruitment were previously published [15,16]. In summary, the study was conducted between November 2007 and April 2010, and recruited adult patients with LRTI that were randomly allocated to receive either 1 g of amoxicillin or placebo three times a day for 7 days.

### 4.2. Data Collection

Data was collected using (a) a case record form (CRF), (b) a symptom diary, and (c) a short version of the diary. The latter was used to collect key outcome variables and AEs during a standardised phone call after 4 weeks, if participants had not returned their diary. For this subgroup analysis, we used information on antibiotic treatment in the previous six months, any medication during the study period, history of regular use of inhaled bronchodilators, steroids, antihypertensive/diuretics, benzodiazepines/antidepressant, oral non-steroidal anti-inflammatory drugs, or influenza vaccination recorded by the responsible clinician in the CRF. The symptom diary was completed by the patient every day from day one, i.e., day of consultation and inclusion, until resolution of symptoms, up to a maximum of 28 days. This diary has previously been validated, is sensitive to change, and internally reliable [23]. Specific AEs, such as diarrhoea, nausea, and rash, were recorded at the end of week one and week two, and over-the-counter (OTC) treatment was recorded on day one. Anxiety and depression related questionnaires were completed by patients on the day of consultation (day one), and at the end of every week for four weeks. For the purpose of this study, we used anxiety and depression reported on day one. All information was collected blind to treatment allocation. All data collection forms were translated into relevant local languages and back-translated to ensure consistency.

### 4.3. Outcomes in the Study

The primary outcomes include the presence of specific AEs, diarrhoea, nausea, and rash. We also created another dichotomous outcome variable for the presence of “any reported AE” for those who had reported either diarrhoea or nausea or rash at the end of week one. Amoxicillin was administered for seven days in the study, and all AEs reported during week one were included in the primary outcome. Subsequent reported AEs was excluded from the analysis.

### 4.4. Sample Size Calculation

As this is a secondary analysis of previously collected data, sample size calculations are no longer relevant. Considering the proportion of any adverse events caused by amoxicillin [15], a subgroup sample size of 136 patients allows detection of a 15% absolute difference in AEs (17.5% versus 2.5%, with 80% power, α = 0.05 (G* power Version 3.1.9.2). Similarly, 302 patients would allow the detection of a 10% absolute difference. Analyses in smaller subgroups were considered underpowered, and were only reported for comprehensive purposes.

### 4.5. Statistical Analysis

The subgroup analyses of the AEs were not pre-specified. For any and each specific AE, we estimated the effect of amoxicillin using logistic regression analysis in Stata (version 13). Subgroup analyses were performed separately for any and each specific AE. The interaction between a particular subgroup (for example, males) and the intervention (in this case amoxicillin) concerns the difference in AEs (of amoxicillin) among the patients in that particular group (males), compared to patients who are not (females). The interaction term is the variable introduced into the statistical model to allow estimation of the size of that difference. The odds ratio in the subgroups estimates the difference in AEs between patients on amoxicillin and those on placebo. The specific subgroups were gender (male/female), age (60 years and older/less than 60 years), and yes/no groups for current and ever smoking, depression/anxiety on the day of the consultation, over-the-counter (OTC) treatment before consultation, antibiotics used in previous six months, use of any medication other than study medication, use of oral bronchodilators, on regular oral or inhaled steroids, on antihypertensive/diuretics, on antidepressant/benzodiazepams, on non-steroidal anti-inflammatory drugs and vaccinated against influenza. Sensitivity analysis was performed by recoding missing information on an AE as the absence of that AE.

### 4.6. Role of Funding Source

GRACE was funded by the European Commission’s Framework Programme 6 (LSHM-CT-2005-518226). The work reported in this publication has been financially supported through TRACE (Translational Research on Antimicrobial resistance and Community-acquired infections in Europe; www.esf.org/trace). The researchers are independent of all funders.

### 4.7. Ethical Approval

Ethical approval for the United Kingdom was granted by Southampton and South West Hampshire Local Research Ethics Committee (B) (ref. 07/H0504/104). Competent authority approval for the UK was granted by the Medicines and Healthcare Products Regulatory Agency. Ethical and competent authority approval was obtained from each local organisation at every research site outside of the UK. Patients who fulfilled the inclusion criteria were given written and verbal information, and informed consent was obtained before enrolment.

## 5. Conclusions

This subgroup analysis provides some evidence that the observed increased risk of any AE or diarrhoea due to amoxicillin was not specific, or more pronounced, in any subgroup of patients. In other words, all adult LRTI patients prescribed antibiotics are likely to be at the same risk for any AE or diarrhoea. We can reiterate the conclusion of the previous GRACE trial that the results do not suggest the use of amoxicillin where only little benefit has been observed for patients with uncomplicated LRTI in primary care. Before prescribing an antibiotic, their potential benefits and harms should be discussed with patients.

## Figures and Tables

**Table 1:** Any adverse event (diarrhoea or nausea or rash) in the whole cohort, and in subgroups of adult patients in the first week after presenting to primary care with a LRTI and allocation to amoxicillin or placebo.

	Amoxicillin	Placebo	Interaction Term (95% CI)	OR for Subgroups (95% CI)
Whole cohort	219/1038	173/1023		1.31 (1.05–1.64) *
Aged 60 years and older	57/292	42/303	1.21 (0.72–2.00)	1.50 (0.97–2.33)
Male	78/413	63/423	1.02 (0.64–1.62)	1.33 (0.92–1.91)
Current smoking	60/307	38/272	1.18 (0.70–1.96)	1.49 (0.95–2.33)
Ever being a smoker	123/559	90/539	1.16 (0.74–1.81)	1.41 (1.04–1.90) *
Depression/anxiety on the day of consultation	77/231	60/239	1.17 (0.72–1.90)	1.49 (0.99–2.22)
OTC treatment before consultation	152/535	112/521	1.28 (0.79–2.07)	1.44 (1.09–1.91) *
Antibiotic used in previous six months	26/143	27/140	0.66 (0.35–1.27)	0.93 (0.51–1.69)
Any medication other than study medication	145/679	115/663	0.94 (0.59–1.51)	1.29 (0.98–1.69)
Oral bronchodilators	20/96	20/108	0.86 (0.41–1.79)	1.16 (0.57–2.31)
On regular oral or inhaled steroids	17/84	18/87	0.71 (0.32–1.56)	0.97 (0.46–2.04)
Antihypertensive/Diuretics	52/239	43/240	0.95 (0.57–1.60)	1.27 (0.81–1.99)
Antidepressant/benzodiazepams	24/98	21/104	0.96 (0.48–1.96)	1.28 (0.65–2.49)
Non-steroidal anti-inflamatory drugs	18/77	16/97	1.19 (0.54–2.61)	1.54 (0.72–3.27)
Influenza vaccine	42/226	31/225	1.10 (0.63–1.95)	1.43 (0.86–2.36)

* Significant at *p*-value < 0.05 and OTC = over-the-counter.

**Table 2:** Diarrhoea in the whole cohort and in subgroups of adult patients in the first week after presenting to primary care with a LRTI and allocation to amoxicillin or placebo.

	Amoxicillin	Placebo	Interaction Term (95% CI)	OR for Subgroups (95% CI)
Whole cohort	129/1038	92/1023		1.43 (1.08–1.90) *
Age 60 years and older	33/242	20/270	1.50 (0.77–2.94)	1.97 (1.09–3.54) *
Male	49/335	37/343	0.94 (0.53–1.69)	1.42 (0.98–2.24)
Current smoking	38/237	18/214	1.57 (0.79–3.11)	2.07 (1.15–3.76) *
Ever being a smoker	76/443	46/443	1.54 (0.86–2.74)	1.79 (1.21 –2.65) *
Depression/anxiety on the day of consultation	39/224	32/239	0.89 (0.48–1.65)	1.36 (0.82–2.26)
OTC treatment before consultation	91/523	55/516	1.63 (0.89–2.99)	1.76 (1.23–2.53) *
Antibiotics in previous six months	15/117	12/110	0.79 (0.33–1.88)	1.20 (0.53–2.69)
Any medication other than study medication	82/551	63/544	0.75 (0.41–1.38)	1.33 (0.94–1.89)
Oral bronchodilators	12/82	10/89	0.91 (0.35–2.36)	1.35 (0.55–3.33)
On regular oral or inhaled steroids #	10/71	9/75	0.80 (0.29–2.21)	1.20 (0.46–3.16)
Antihypertensive/Diuretics	38/198	20/211	1.79 (0.92–3.49)	2.27 (1.27–4.05) *
Antidepressant/benzodiazepams	13/81	8/87	1.32 (0.49–3.54)	1.89 (0.74–4.82)
Non-steroidal anti-inflamatory drugs #	13/60	4/81	4.02 (1.19–13.55) *	5.32 (1.64–17.29) *
Influenza vaccine	29/184	17/194	1.43 (0.70–2.92)	1.95 (1.03–3.68) *

* Significant at *p*-value < 0.05 and OTC = over-the-counter; # variables underpowered.

**Table 3:** Nausea in the whole cohort and in subgroups of adult patients in the first week after presenting to primary care with a LRTI and allocation to amoxicillin or placebo.

	Amoxicillin	Placebo	Interaction Term (95% CI)	OR for Subgroups (95% CI)
Whole cohort	99/1038	82/1023		1.21 (0.89–1.64)
Age 60 years and older	26/243	24/270	1.01 (0.51–2.02)	1.22 (0.68–2.20)
Male	26/336	26/342	0.77 (0.39–1.52)	1.02 (0.58–1.79)
Current smoking	25/237	20/213	0.90 (0.44–1.85)	1.14 (0.61–2.11)
Ever being a smoker	48/444	43/441	0.84 (0.45–1.56)	1.12 (0.73–1.73)
Depression/anxiety on the day of consultation	37/224	31/237	0.09(0.57–2.09)	1.31 (0.78–2.20)
OTC treatment before consultation	68/523	57/514	0.93 (0.47–1.82)	1.19 (0.82–1.74)
Antibiotics in previous six months	14/117	13/110	0.80 (0.33–1.92)	1.01 (0.45–2.26)
Any medication other than study medication	66/552	54/542	1.00 (0.52–1.93)	1.23 (0.84–1.79)
Oral bronchodilators	13/82	8/89	1.65 (0.61–4.45)	1.91 (0.75–4.87)
On regular oral or inhaled steriods #	10/71	9/75	0.98 (0.35–2.71)	1.20 (0.46–3.16)
Antihypertensive/Diuretics	21/198	20/211	0.91 (0.43–1.89)	1.13 (0.59–2.16)
Antidepressant/benzodiazepams	13/81	13/87	0.87 (0.35–2.14)	1.09 (0.47–2.51)
Non-steroidal anti-inflamatory drugs #	9/60	9/81	1.16 (0.41–3.30)	1.41 (0.52–3.80)
Influenza vaccine	18/185	14/194	1.17 (0.52–2.62)	1.38 (0.67–2.87)

* Significant at *p*-value < 0.05 and OTC = over-the-counter; # variables underpowered.

**Table 4:** Rash in the whole cohort and in subgroups of adult patients in the first week after presenting to primary care with a LRTI and allocation to amoxicillin or placebo.

	Amoxicillin	Placebo	Interaction Term (95% CI)	OR for Subgroups (95% CI)
Whole cohort	37/1038	33/1023		1.11 (0.69–1.79)
Age 60 years and older	14/242	7/269	2.77 (0.93–8.23)	2.29 (0.91–5.79)
Male	16/336	6/341	3.72 (1.22–11.36) *	2.79 (1.08–7.22) *
Current smoking	8/237	5/213	1.36 (0.39–4.75)	1.45 (0.47–4.51)
Ever being a smoker	22/444	14/442	2.06 (0.78–5.46)	1.59 (0.80–3.16)
Depression/anxiety on the day of consultation	19/224	11/238	2.44 (0.90–6.58)	1.91 (0.89–4.11)
OTC treatment before consultation	25/522	25/514	0.64 (0.22–1.87)	0.98 (0.56–1.74)
Antibiotics in previous six months	7/117	6/110	0.98 (0.28–3.42)	1.10 (0.36–3.39)
Any medication other than study medication	25/551	20/543	1.33 (0.49–3.64)	1.24 (0.68–2.26)
Oral bronchodilators	2/81	4/89	0.45 (0.07–2.72)	0.54 (0.09–3.02)
On regular oral or inhaled steriods #	2/71	2/75	0.94 (0.12–7.32)	1.06 (0.14–7.72)
Antihypertensive/Diuretics	9/197	7/210	1.33 (0.42–4.17)	1.39 (0.50–3.80)
Antidepressant/benzodiazepams	6/80	6/87	0.96 (0.27–3.49)	1.09 (0.34–3.54)
Non-steroidal anti-inflamatory drugs #	2/60	6/81	0.34 (0.06–1.89)	0.43 (0.08–2.21)
Influenza vaccine	6/184	4/194	1.53 (0.38–6.11)	1.60 (0.44–5.77)

* Significant at *p*-value < 0.05 and OTC = over-the-counter; # variables underpowered.
